# Evaluator standpoint and ecological context shape psychological momentum measurement in sport

**DOI:** 10.3389/fspor.2026.1917087

**Published:** 2026-08-21

**Authors:** Yusuke Asai, Noriyuki Kida

**Affiliations:** 1 Department of Sport Cultural Studies, Hokkaido University of Education, Hokkaido, Japan; 2 Graduate School of Science and Technology, Kyoto Institute of Technology, Kyoto, Japan; 3 Faculty of Arts and Sciences, Kyoto Institute of Technology, Kyoto, Japan

**Keywords:** actor-experienced momentum, affordances, ecological validity, embodied cognition, observer inference

## Abstract

Psychological momentum (PM) has been widely discussed in sport psychology, yet the term has been used to describe different levels of evidence, including objective performance sequences, observer judgments, retrospective reconstructions, simulated experiences, and real-time reports from athletes. This conceptual ambiguity makes it difficult to determine whether previous studies have examined the same construct, different components of a broader PM process, or related but distinct phenomena. The present conceptual analysis clarifies how PM-related evidence has been obtained in sport research and how such evidence should be interpreted according to evaluator standpoint and ecological context. First, major theoretical accounts of PM are reviewed, including PM as psychological force, an antecedent–consequence process, a dynamic system, persistence and carryover of mental experience, and objective performance dynamics. Building on this multilevel view, the article proposes a framework organized around two axes: evaluator standpoint and access to first-person PM experience, and the ecological representativeness of the assessment context. This framework distinguishes third-person or other-perceived PM, vicarious or simulated actor PM, reconstructed actor PM, and actor-experienced PM. Within the actor pathway, objective events acquire psychological meaning through expectation-based embodied appraisal, while affective and bodily states may operate recursively as both inputs to and consequences of appraisal. The article argues that laboratory/scenario-based, video-based, and live-competition-based approaches should not be treated as competing methods, but as complementary approaches that provide different kinds of PM-related evidence. Clarifying these distinctions can improve construct validity in PM research and support more precise interpretation of findings across experimental, video-assisted, and field-based studies.

## 1 Introduction

The purpose of this paper is to discuss how psychological momentum (PM) has been assessed in sport and how evaluator standpoint should be considered in PM assessment. Since the 1980s, PM has attracted scholarly attention and has been investigated primarily from psychological perspectives. However, although the relationship between the evaluator and the target match should be important for understanding PM assessment, this relationship has rarely been discussed in detail. One reason for this may be that PM is a diverse and multilevel construct. Therefore, this paper first clarifies the multilevel nature of PM and the difficulty of interpreting PM-related evidence. It then argues that, precisely because of this conceptual complexity, it is necessary to disentangle the relationship between the evaluator, the target event, and the ecological context in which PM is assessed. In doing so, the paper highlights the importance of treating PM as a multilevel phenomenon and of bridging experimental and field-based approaches in PM research.

### 1.1 The multilevel nature of psychological momentum

Before discussing the relationship between the evaluator, the target match, and the ecological context of PM assessment, it is necessary to clarify how PM is understood as a construct. This is because objective performance sequences, observer judgments, retrospective reconstructions of past experience, and real-time reports from athletes during competition may all provide evidence related to PM, but they do not necessarily access the same level of the PM process. Without clarifying the conceptual foundations of PM, it is difficult to determine whether different assessment approaches measure the same construct, capture different components of a broader construct, or address related but distinct phenomena. Therefore, this paper first reviews major theoretical accounts of PM and then discusses how PM assessment differs according to ecological context and evaluator standpoint.

Definitions of PM are not uniform, and different aspects have been emphasized across research traditions. In this paper, major theoretical accounts of PM are organized in terms of PM as psychological force, PM as an antecedent–experience–consequence process and multidimensional chain, PM as a dynamic system, PM as persistence and carryover of mental experience, and momentum-related performance dynamics as objective evidence. Through this organization, PM is positioned not as a single directly observable state, but as a multilevel construct composed of several interrelated levels of evidence.

### 1.2 PM as psychological force

Iso-Ahola provided an important theoretical foundation in the early development of PM research. Iso-Ahola and Mobily (1) defined PM as “an added or gained psychological power which changes a person's view of himself or of others or others’ view of him and of themselves” (p. 392). In this definition, PM is not simply a scoring pattern or performance sequence. Rather, it is conceptualized as a psychological force that changes self-perception, perception of others, or perceptions of how one is viewed by others.

Based on Iso-Ahola and Blanchard (2) and Iso-Ahola and Dotson (3), PM in this tradition can be understood as a psychological advantage generated by early success. It alters self-perception, opponent perception, confidence, effort, and performance, thereby increasing the likelihood of subsequent success. In brief, Iso-Ahola's line of work conceptualizes PM as a psychological process through which the perception of success increases the perceived likelihood of further success.

### 1.3 PM as antecedent–experience–consequence/multidimensional chain

Vallerand et al. (4) defined PM as “a perception that the actor is progressing toward his/her goal” (p. 94) and proposed the antecedents–consequences psychological momentum model. In this model, situational and personal antecedents elicit the perception of PM, which then influences actual performance through contextual and personal consequences. Thus, Vallerand et al. (4) conceptualized PM as the perception of moving toward a goal and argued that this perception affects subsequent performance inferences and performance.

Taylor and Demick's (5) multidimensional model further elaborated this process. In their model, after a precipitating event occurs, the way in which the event is experienced leads to changes in cognition, affect, and physiology. These changes then influence behavior and, ultimately, performance and outcome. In this sense, Vallerand et al. (4) and Taylor and Demick (5) positioned PM not as a single affective state, but as a chain-like process involving events, experience, psychological states, behavior, and performance.

It should be noted, however, that the theories proposed by both Vallerand et al. (4) and Taylor and Demick (5) are fundamentally process models, and the notion that PM perception actualizes performance enhancement is left as a theoretical assumption rather than an empirically proven fact.

### 1.4 PM as dynamic system

Gernigon et al. (6) introduced a dynamic systems perspective into PM research. Drawing on Adler (7), Vallerand et al. (4), and Taylor and Demick (5), Gernigon et al. (6) conceptualized PM as psychological dynamics and explained it in terms of key characteristics of dynamic systems, including nonstationarity, nonlinearity, history dependence, and hysteresis.

Importantly, Gernigon et al. (6) did not treat the score situation itself as PM. Rather, they manipulated score progression as a control parameter and examined how PM-related psychological variables, such as anxiety, confidence, and achievement goals, changed in response to that score progression. Thus, in this line of research, PM is not understood as a fixed psychological state, but as a history-dependent process that changes through the interaction of time, preceding events, score progression, and psychological responses. This theoretical perspective has been further developed in studies by Briki et al. (8–11) and Den Hartigh and Gernigon (12).

In summarizing the preceding discussion of PM in sport, Vallerand et al. (4), Taylor and Demick (5), and Gernigon et al. (6) would likely share the view that PM involves a chain-like process. However, the former accounts can be understood as approaching PM from a more micro-level perspective, whereas the latter conceptualizes PM from a more meso- or macro-level perspective. At the same time, because these processes may also be cyclical, Iso-Ahola and Dotson's (3) account can likewise be regarded as sharing the idea of a chain-like process, in the sense that success gives rise to further success. Thus, if these accounts are to be distinguished, the difference lies not in whether they conceptualize PM as a chain-like process, but in the theoretical mechanisms assumed to operate within that process.

### 1.5 PM as persistence and carryover of mental experience

The sport PM literature reviewed above has primarily treated PM in relation to success and failure, score progression, movement toward or away from a goal, changes in psychological states, and effects on performance. However, it is also important to consider a broader psychological explanation of why previous events continue to influence subsequent cognition, affect, judgment, and action. To address this issue, it is useful to refer to general psychological accounts of PM beyond the sport context.

Honey et al. (13) defined PM as “the lingering of contentful mental states over several minutes, even in the absence of external cues or goals” (p. 285). Here, contentful mental states refer to mental states that are related to specific features of a recent experience. Honey et al.'s (13) account does not replace sport-specific PM models, but it is useful for understanding PM as a phenomenon in which recent experiences persist and carry over into subsequent thoughts, emotions, and action tendencies.

From this perspective, points scored, errors, tactical successes, and opponent dominance in sport can be understood not as isolated events that disappear immediately after they occur, but as experiences that may carry over into subsequent confidence, attention, affect, perceived action possibilities, and decision-making. Therefore, Honey et al.'s (13) general psychological account provides a complementary perspective for explaining history dependence and the persistence of experience in sport PM.

### 1.6 Momentum-related performance dynamics as objective evidence

The preceding accounts conceptualize PM primarily as a psychological process involving perceived progress toward or away from a goal, changes in cognitive, affective, bodily, motivational, and behavioral states, dynamic history-dependent fluctuations, and the carryover of recent mental experience. However, PM research has also used objective performance sequences, such as scoring runs, winning streaks, point sequences, and other temporally ordered events, as indicators or models of momentum (14, 15). These approaches should not be equated with athletes’ subjective PM experience. Nevertheless, they are relevant because they describe the event structure from which PM may emerge, be inferred by observers, or be reconstructed retrospectively by athletes.

In addition to raw event sequences, objective momentum dynamics can also be modeled using performance indicators that compare observed performance with expected or contextually valued performance. For example, momentum may be represented as a dynamically updated deviation between recent performance and an expected performance trajectory, with more recent deviations receiving greater weight than earlier deviations. Such approaches may use scoring sequences, point sequences, expected performance, expected points, expected goals, turnover sequences, opponent strength, shot quality, or action-value indicators to estimate whether recent performance is better or worse than expected (14, 16). These models do not measure subjective PM experience directly. However, they can describe the objective performance trajectory to which athletes, coaches, spectators, and analysts may respond. They can also be compared with observer-inferred PM, reconstructed actor PM, or actor-experienced PM to examine whether subjective or inferred momentum corresponds to, anticipates, or diverges from objective event dynamics.

One recent example is the exponential rate of change (ERC) indicator proposed by Jekauc et al. (17). The ERC represents recent performance dynamics as a recency-weighted accumulation of deviations between observed and expected performance, such as actual versus expected points. Earlier deviations are discounted exponentially, so that recent events contribute more strongly to the current value, while previous events retain a progressively diminishing influence. This logic is consistent with performance-analytic approaches that assign greater weight to recent observations (18, 19) and with psychological accounts suggesting that current affective and evaluative responses are influenced by the rate of progress relative to a reference value or goal and by the persistence of recent mental experience (13, 20, 21). The ERC should therefore be interpreted as an objective indicator of momentum-related performance dynamics that can be compared with subjective or observer-inferred PM, rather than as a direct measure of psychological PM itself. From an applied perspective, recency-weighted indicators such as the ERC may help identify acute negative performance dynamics that precede or coincide with critical organizational decisions, including coach dismissals, and may therefore serve as potential early-warning signals, although their prospective predictive validity remains to be established (17). The specific decay parameter used in an ERC model should be regarded as an operational assumption that requires validation across sports and temporal scales.

This issue is also related to the concept of strategic momentum. Although relatively few studies in sport have explicitly examined strategic momentum, Gauriot and Page (22) defined it as follows: “It is purely determined by the state variables of the competition (e.g., scoreline, time left to play) and, typically, whether a player is ahead or behind. It is not path dependent in that it is not influenced by how this state was reached” (p. 138). Strategic momentum can therefore be understood as an objective, game-state-based form of momentum that reflects which side has an advantage at a given moment in a contest, based on variables such as scoreline and time remaining. In this sense, strategic momentum overlaps with objective performance dynamics insofar as both concern objective indicators of the competitive situation. However, the non-path-dependent nature of strategic momentum also marks a clear distinction between strategic momentum and PM. Whereas strategic momentum is determined by the current state of the competition, PM is generally conceptualized as a process shaped by prior events, perceived progress, psychological responses, and the carryover of recent experience.

**Figure 1:**
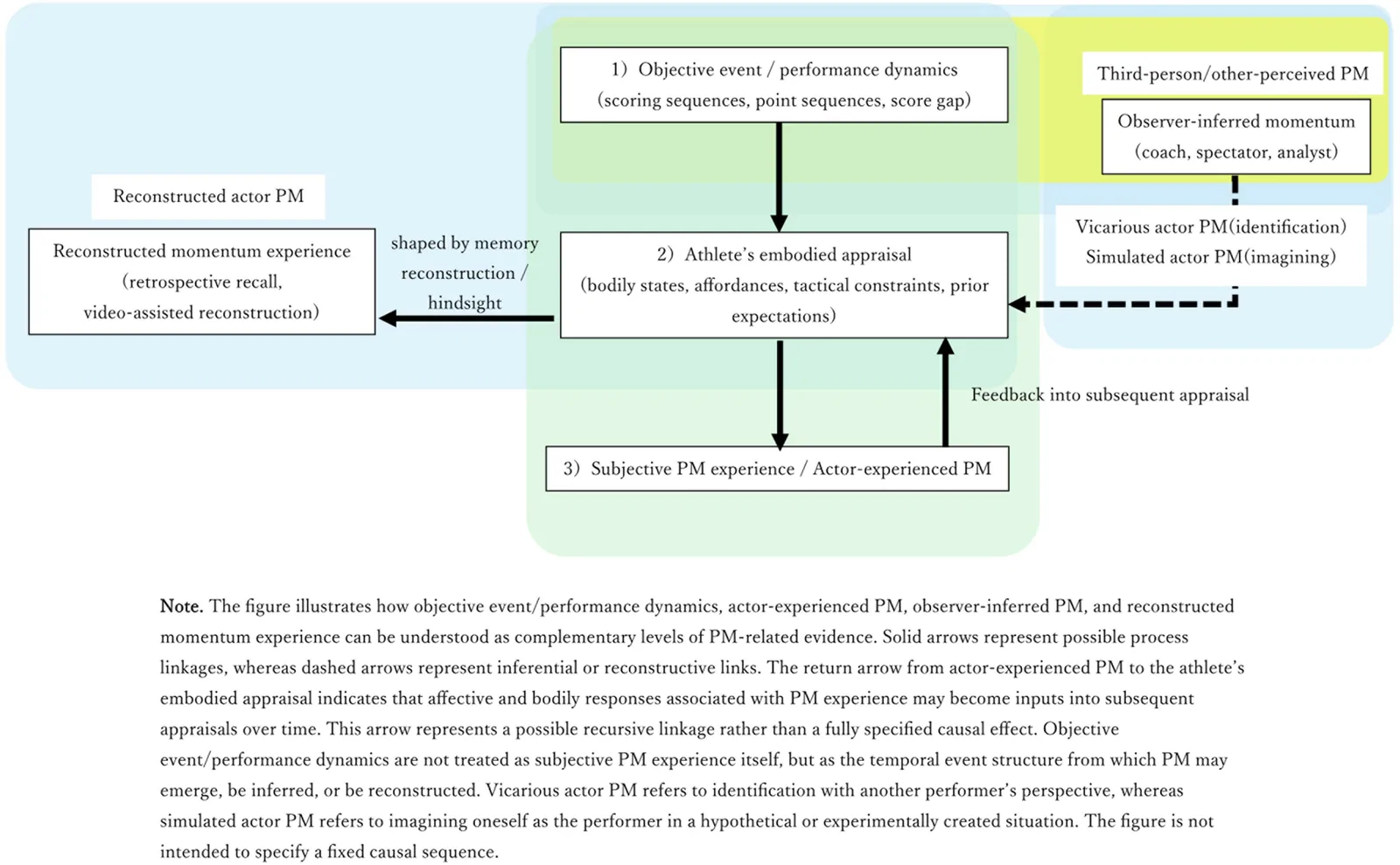
A multilevel framework linking objective event dynamics, actor-experienced PM, observer-inferred PM, and reconstructed momentum experience.

Within the present framework, strategic momentum should be treated as a related but distinct, non-path-dependent form of objective game-state evidence rather than as a subtype or direct measure of psychological PM. In Figure 1, it can therefore be positioned within Level 1 alongside other objective event/performance dynamics. At the same time, the current game state may function as a contextual input to athletes’ expectation-based embodied appraisal because its psychological meaning depends on how scoreline, time remaining, and competitive advantage are interpreted in relation to goals, prior expectations, tactical intentions, and perceived controllability. Strategic momentum may therefore contribute to the conditions under which PM emerges, is inferred, or is reconstructed, but it should not be equated with subjective PM experience.

### 1.7 Integrative perspective: toward a multilevel framework for PM assessment

Taken together, these accounts suggest that PM should not be treated as a single directly observable state. Rather, PM can be understood as a multilevel, temporally extended process in which objective performance events, athletes’ appraisals of goal progress, cognitive, affective, bodily, motivational, and behavioral changes, carryover from recent experience, and subsequent performance are interrelated over time.

In this way, the theoretical foundations relevant to PM, including psychological force, cognitive and information-processing approaches, dynamic systems perspectives, and unconscious carryover as a general property of human mental life, are all anchored in the first-person standpoint. That is, these theories presuppose that an athlete or human actor personally experiences success, a triggering event, temporally ordered success or failure, or even the lingering effects of an activity such as reading a novel. These theoretical accounts assume that PM emerges through the actor's own cognition, appraisal, or experience of such events.

However, many empirical studies conducted to date have not obtained data from situations in which participants themselves actually succeed or fail during performance. Instead, they have often relied on simulated experiences, inferences, or vicarious and retrospective reconstructions of success, failure, and momentum-related situations. In this context, the appropriate relationship between the research setting, the participant, and the target performance situation may depend on what the study aims to clarify: the nature of PM itself, the relationship between PM and human cognition, or the relationship between PM and subsequent performance.

Despite this issue, systematic theoretical accounts of PM remain relatively limited, with notable exceptions such as Iso-Ahola and Dotson (3) and Briki (23). These accounts have also primarily focused on the nature of PM itself. Therefore, the present article next clarifies the relationship between the participant's standpoint and the context of the research environment. By doing so, it explains what can be inferred from data obtained in each type of environment, while taking into account the relationship between PM and the participant who evaluates or experiences it.

Before explaining the specific categories shown in Table 1, it is necessary to clarify why the two axes were adopted. These axes were derived from recurring methodological differences in previous PM studies rather than from a single theoretical model. However, they can be theoretically supported by two complementary considerations: actor–observer asymmetry (24) and representative learning design (25).

**Table 1:** Classification of human-based PM assessment approaches by ecological context and access to athlete experience.

		Continuum of ecological representativeness		
		Laboratory/scenario-based PM	Recorded real-match video-based PM	Live-competition-based PM
Evaluator standpoint and access to first-person PM experience	Third-person/other-perceived PM	Vallerand et al. (4) Miller and Weinberg (26) Burke et al. (27) Briki et al. (11)†	Gautier et al. (39)	Burke et al. (28)
	Vicarious/simulated actor PM	Gernigon et al. (6) Briki et al. (11)† Den Hartigh and Gernigon (12)	Den Hartigh et al. (41)	
	Reconstructed actor PM		Briki et al. (8) Briki et al. (9)	
	Actor-experienced PM	Perreault et al. (29) Stanimirovic and Hanrahan (40) Briki et al. (10)		Iso-Ahola and Blanchard (2) Asai et al. (31)

Note. Table 1 is organized around two axes. The left-side axis label, “Evaluator standpoint and access to first-person PM experience,” applies to the four evaluator-standpoint categories shown in the colored category cells: third-person/other-perceived PM, vicarious/simulated actor PM, reconstructed actor PM, and actor-experienced PM. The top axis label, “Continuum of ecological representativeness,” applies to the three ecological assessment contexts: laboratory/scenario-based PM, recorded real-match video-based PM, and live-competition-based PM. Colors are used as visual aids to distinguish the degree and type of access to first-person PM experience: yellow indicates third-person/other-perceived PM, light blue indicates vicarious/simulated actor PM and reconstructed actor PM, and green indicates actor-experienced PM. This color scheme corresponds to that used in Figure 1. Some studies were classified into more than one category when they included multiple participant perspectives or measurement procedures. Diagonal hatching indicates combinations that are not applicable under the definitions adopted in the present framework, whereas unhatched empty cells indicate that no representative study was identified for the relevant combination among the studies reviewed here and should not necessarily be interpreted as indicating that the combination is theoretically impossible. In the present framework, reconstructed actor PM presupposes the retrospective reconstruction of the actor's own prior experience. It therefore does not apply to purely hypothetical scenario-based assessments, although it could, in principle, follow a laboratory-based performance. Objective event/performance dynamics are not included as a row because they do not necessarily involve a human evaluator standpoint; instead, they are represented in Figure 1 as a distinct level of PM-related evidence. The dagger symbol (†) indicates that a study could reasonably be interpreted as belonging to two overlapping categories and was therefore included in both categories.

The first axis concerns the standpoint of the evaluator and the degree of access to the athlete's first-person PM experience. This axis is informed by actor–observer asymmetry. Jones and Nisbett (24) argued that actors and observers differ in terms of information availability, information processing, and salience. Actors have direct access to their own intentions, emotions, bodily states, and situational concerns, whereas observers do not have direct access to the actor's internal states or personal history. Observers must therefore infer the actor's state from externally available information, such as visible behavior, performance outcomes, or situational cues.

In addition, actors and observers differ in what becomes salient. For actors, attention is often directed toward the external situation, including the environmental and situational cues that elicit and guide action. For observers, by contrast, the actor and the actor's behavior tend to become the most salient objects within the situation. Applied to PM research, this means that athletes may evaluate PM through intentions, bodily states, affective changes, tactical expectations, and perceived action possibilities, whereas observers may infer PM from score changes, performance sequences, and visible behavioral cues. For this reason, evaluator standpoint and access to first-person experience constitute an important axis for classifying PM-related approaches.

The second axis concerns the ecological representativeness of the assessment context. Drawing on representative learning design (25), this axis refers to the extent to which the research context preserves the informational constraints, temporal demands, perception–action couplings, interpersonal constraints, and action possibilities of the target performance environment. In PM research, this distinction is important because PM is not only a judgment about success or failure, but a process that may emerge while athletes are acting under time pressure, bodily involvement, tactical demands, and interpersonal opposition.

Importantly, laboratory- or scenario-based studies and video-based studies are not treated here as ecological in the same sense as live competition. Rather, they are positioned along a continuum of ecological representativeness. Laboratory- and scenario-based studies provide strong experimental control and standardization, and they are useful for examining general tendencies or main effects under controlled conditions. However, they usually preserve fewer of the bodily, tactical, interpersonal, and consequential constraints of actual competition. Video-based studies may preserve the temporal and visual structure of real match events more strongly, but participants are still separated from real-time action, bodily involvement, and performance consequences. Live-competition-based studies preserve these constraints more directly, although they are less experimentally controllable.

This classification should not be understood as opposing ecological perspectives to cognitive or information-processing approaches. Cognitive and information-processing approaches remain useful for explaining how athletes interpret success, failure, score changes, and goal progress. The ecological-representativeness axis instead clarifies the context in which such interpretations are elicited and the extent to which that context preserves the informational and action constraints of actual sport performance. Therefore, crossing evaluator standpoint with ecological representativeness helps clarify what kind of PM-related evidence each approach can provide and how findings from different research contexts should be interpreted.

## 2 Evaluator standpoint and ecological context in PM assessment

### 2.1 Clarifying levels of PM-related evidence: observer inference, reconstructed experience, and actor experience

Previous PM research was developed through several innovative methodological approaches. Gernigon et al. (6) and Briki et al. (8, 9, 11) played pioneering roles in examining PM's dynamic nature. However, these studies also highlight an important methodological issue: PM studies have not always accessed the same level of PM-related evidence. For example, Briki et al. (11) asked participants in the observer condition to infer PM from another performer's situation, whereas Gernigon et al. (6) asked participants to imagine themselves as actors in simulated performance situations. Other studies reconstructed athletes’ previous PM experiences from video footage of natural sport situations (8, 9). These approaches are valuable; however, they should be distinguished from the real-time measurements of actor-experienced PM obtained from athletes during actual athletic performances.

This distinction is important because video- and scenario-based PM studies differ in the degree to which they provide access to the athlete's first-person experiences. For example, Vallerand et al. (4), Miller and Weinberg (26), Burke et al. (27, 28), and the observer condition in Briki et al. (11) asked participants, spectators, or observers to evaluate PM based on scenarios, match situations, or performance information involving athletes or teams with whom they were not directly involved. These studies can be considered as measuring third-person or other-perceived PM because participants infer momentum from externally available information such as score changes, performance sequences, and visible behavioral cues. In contrast, Gernigon et al. (6), the virtual actor condition in Briki et al. (11), and Den Hartigh and Gernigon (12) asked participants to imagine themselves as athletes in simulated or vicarious performance situations. Such studies are closer to actor-centered PM, but the actors’ perspectives remain hypothetical because participants are not actually exposed to the bodily, tactical, and interpersonal constraints of real competition.

Further distinction is required in video-assisted recall studies. Briki et al. (8, 9) used athletes’ previous competitive performances to reconstruct PM experiences. This approach is not simply observer-based because athletes evaluate their own past experiences rather than the performance of another athlete. However, these assessments involved reconstruction of past momentum experience and may have been susceptible to memory reconstruction or hindsight and outcome bias. Therefore, such studies may be more accurately described as reconstructed-actor PM rather than as direct actor-experienced PM.

In contrast, actor-experienced PM refers to PM measured using individuals engaged in a task or competition. For example, Perreault et al. (29) and Briki et al. (10) asked participants to perform cycling tasks in experimentally controlled competitive environments and report their PM while physically involved in the task. These studies are important because they allow participants to evaluate PM relative to their bodily state, effort, fatigue, perceived progress toward the goal, and relative position against the opponent. In this way, investigating actor-experienced PM in a controlled experimental environment can be regarded as an important research context for obtaining theoretically grounded data, because it allows researchers to collect first-person data from actors while taking into account the characteristics of the sport or task.

The distinction between observer-based and actor-experienced PM can be theoretically grounded in actor–observer asymmetry (24) because actors and observers do not have equal access to the same information (30). Actors can draw on their own intentions, goals, bodily sensations, emotional states, tactical expectations, and personal histories, whereas observers can only infer these states from external information. Therefore, PM ratings can differ depending on whether the evaluator is an external observer, an athlete engaged in actual competition, or a simulated or reconstructed actor. However, this does not imply that the observer-based PM studies are invalid. Rather, they provided measurements of observer-inferred PM based on observable performance patterns.

PM-related assessment approaches also differ in their ecological context. Although laboratory/scenario-based, recorded-video-based, and live-competition-based studies preserve different degrees of experimental control and actual performance constraints, live-competition-based actor-experienced PM remains underrepresented in the current literature. If PM is understood as an embodied and situated psychological process, real-time field assessment may provide more direct access to athletes’ ongoing subjective experience than observer-based or retrospective approaches, while also introducing the measurement limitations discussed below.

A recent real-time volleyball study illustrated the feasibility of this approach (31). In this study, players verbally recorded PM fluctuations during the natural intervals between rallies in actual practice matches. The findings showed that PM changes depended not only on whether a point was won or lost but also on play content, game stage, and player expertise, which suggested that PM is shaped by athletes’ situated interpretation of unfolding match events. This evidence supports the need to distinguish between live actor-experienced PM and observer-inferred, simulated, or retrospectively reconstructed PM.

However, real-time athlete reports are not free from measurement limitations. Repeated *in-situ* assessments may alter athletes’ self-monitoring or attentional focus and thereby produce measurement reactivity, while brief ratings may also contain response noise or disproportionately reflect the immediately preceding event (32, 33). Evidence from concurrent verbal-report research further suggests that reactivity depends on the reporting procedure: simple concurrent verbalization may be less reactive than directed requests to explain ongoing thoughts, although reactivity cannot be ruled out entirely (34). In competitive sport, athlete self-reports may also be influenced by strategic or self-presentational concerns, depending on how the information is used and who has access to it (35). Accordingly, real-time reports may provide more direct access to ongoing subjective PM experience, but this access should not be regarded as unmediated or error-free.

### 2.2 Assessment contexts and access to athlete experience in PM research

To clarify this methodological issue, PM studies can be classified into two dimensions: the ecological context in which PM is assessed, and the degree of access to the athlete's first-person experience. Table 1 presents representative PM studies by placing laboratory/scenario-based studies, recorded real-match video-based studies, and live-competition-based studies on the horizontal axis. The vertical axis distinguishes studies in which participants observed and evaluated another person's match or performance: studies in which participants were asked to imagine themselves as the actors or were presented with past performances as a stimulus in a reconstructed setting, and studies in which participants actually performed a match or task themselves.

PM assessment research has advanced primarily through laboratory- and scenario-based approaches, which provide experimental control and standardized conditions and remain particularly useful for exploratory work in underexamined sports. However, these approaches provide one form of PM-related evidence and should not be interpreted as fully representing PM across all evaluator standpoints and ecological contexts. The appropriate research design therefore depends on the type of PM-related evidence that a study aims to obtain.

Third-person or other-perceived PM is relatively amenable to large-sample research because participants do not need to be directly involved in the performance situation. They can evaluate momentum simply by being presented with standardized and externally available information, such as scenarios, score changes, performance sequences, video clips, or visible behavioral cues. Because responses can be obtained by presenting the same information to many participants, it is comparatively easier to increase the sample size and examine general tendencies in how momentum is inferred across participants, sports, teams, or competitive situations.

Similarly, vicarious or simulated actor PM can often be investigated through relatively large-sample designs because participants can be asked to imagine themselves as performers in standardized scenarios or experimentally controlled situations. These approaches are useful for examining the robustness and reproducibility of PM-related judgments, but they should not be interpreted as providing direct evidence of ongoing actor-experienced PM during actual competition.

Actor-experienced PM, in contrast, is less easily captured through large-sample standardized designs because it refers to PM as experienced by athletes while they are actually engaged in performance. Such experience is embedded in bodily states, tactical demands, interpersonal constraints, time pressure, and the athlete's own sense of action possibilities. For this reason, actor-experienced PM may often require small-scale, live-competition-based, or otherwise context-sensitive research designs. These designs may face practical limitations, including difficulty in obtaining large sample sizes, but they can provide detailed evidence about how PM emerges, changes, and is experienced in actual performance environments.

Reconstructed actor PM occupies an intermediate position. Because it relies on athletes’ retrospective reconstruction of their own previous PM experiences, often supported by video footage and interview-based inquiry, it may provide richer access to the athlete's standpoint than third-person or simulated approaches. Accordingly, it should be distinguished from simulated actor PM based on purely hypothetical scenario-based assessments, although retrospective reconstruction could, in principle, follow either a laboratory-based performance or a previous performance in actual competition. It may also provide more detailed verbal accounts than real-time actor-experienced PM assessments, because interviews allow athletes to elaborate on what they perceived, attended to, felt, and interpreted at the time. A major strength of this approach is therefore that it allows researchers to examine athletes’ interpretations of their past PM experiences in considerable detail. Through interviews, athletes can describe how they perceived the situation, what they attended to, how they interpreted preceding events, and how they made sense of changes in confidence, control, emotion, bodily state, motivation, or tactical possibilities. In this respect, reconstructed actor PM can generate rich, context-sensitive data that are difficult to obtain from standardized scenarios, observer-based evaluations, or brief real-time ratings during performance. At the same time, reconstructed actor PM is affected by memory, hindsight, and retrospective meaning-making. Therefore, it should not be treated as identical to ongoing actor-experienced PM during actual competition. For this reason, reconstructed actor PM is often well suited to small-scale, case-based, video-assisted qualitative, or mixed-method designs that allow researchers to examine distinctive PM episodes in detail while carefully considering both the richness and the retrospective nature of the data. Therefore, the key issue is not whether large-sample or small-scale research is inherently superior. Rather, PM-related findings should be interpreted according to the type of PM-related evidence being obtained. Third-person, other-perceived, vicarious, and simulated PM may be well suited to large-sample designs that clarify general tendencies and statistical associations. Actor-experienced PM and reconstructed actor PM may be better suited to smaller-scale, context-rich designs that clarify the situated meaning of distinctive PM episodes. Recognizing these methodological affordances and limitations can support a more balanced interpretation of PM research and a more precise understanding of what each type of study can and cannot reveal.

### 2.3 Ecological context and the interpretation of PM-related evidence

Laboratory/scenario-based and live-competition-based approaches should not be understood as competing methods in which one is inherently superior to the other. Rather, they provide access to different kinds of PM-related evidence. Laboratory- and scenario-based approaches are important because they allow researchers to control task conditions, manipulate specific variables, and examine general tendencies or main effects under standardized conditions. Such approaches are therefore useful for clarifying certain properties of PM and for comparing PM-related judgments across participants, sports, or experimental conditions.

At the same time, PM is also a situated and time-sensitive process that may be shaped by the environment in which it occurs. From the perspective of embodied and situated cognition, human cognition is not detached from the body, action, and environment. Wilson (36), for example, argued that cognition is situated and time-pressured. Applied to PM, this means that athletes’ evaluations of momentum may depend not only on score changes or performance sequences, but also on the bodily, temporal, tactical, and interpersonal constraints under which they are acting. Therefore, even when the same match or performance sequence is evaluated, PM-related cognition may differ depending on whether it is assessed in a laboratory/scenario-based context, through video footage, or during live competition.

This distinction can also be understood in terms of affordances and perception–action coupling. In experimental or scenario-based settings, the information available to participants is necessarily constrained by the design of the task. This is not a weakness in itself, because such control is often necessary for examining specific mechanisms or generalizable effects. However, the resulting data should be interpreted in relation to those constraints. In live competition, athletes are embedded in a dynamic environment in which they must continuously select relevant information, respond to opponents and teammates, and act under time pressure and performance consequences. In team sports, shared affordances may be particularly important because teammates’ actions provide information for coordination and influence the action possibilities available at a given moment (37).

Thus, the key point is not that live-competition-based approaches are more valid in all respects. Rather, laboratory/scenario-based, video-based, and live-competition-based approaches differ in the ecological context in which PM-related evidence is obtained. Laboratory/scenario-based approaches may be better suited to examining controlled mechanisms and general tendencies, whereas live-competition-based approaches may be better suited to examining actor-experienced PM as a situated process embedded in actual performance constraints. Treating these approaches as interchangeable forms of PM measurement risks obscuring what kind of PM-related evidence is being accessed. For this reason, PM assessment methods should be interpreted in relation to both the evaluator's standpoint and the ecological context in which the data are obtained.

### 2.4 Experimental control versus field validity: a complementary framework

Building on this complementary view, PM research should further consider how the value of each assessment context depends on the structure of the target sport. Because sports differ in scoring frequency, rule structure, temporal organization, interpersonal interaction, and opportunities for action, the indicators and contexts that are informative for PM-related evidence may also differ across sports. Laboratory/scenario-based studies can be useful for systematically comparing such sport-specific features, whereas video-based and live-competition-based studies can examine whether these features operate similarly in more representative performance contexts.

PM characteristics may also differ across sports. For example, the PM in volleyball may be relatively similar to the PM in badminton or table tennis because these sports share similar rule structures, in which play unfolds across a net separating the two sides of the court. In such sports, it is relatively easy to assume that winning points improves PM, whereas losing points decreases PM. In contrast, basketball, soccer, rugby, and handball share the common feature of moving the ball toward a goal; however, the ease with which points are scored differs substantially across these sports. Therefore, it is unclear whether their PM structures are similar. In these sports, PM may change even without scoring, which suggests that PM is partly dependent on each sport's rule structure.

Baseball and softball may also show similar PM patterns, because their scoring systems are strongly structured by innings. Likewise, swimming and track-and-field sprint events, or middle- and long-distance events, may show sport-specific similarities in PM. From this perspective, it is important for PM research development to examine, under experimental conditions, whether sports with similar rules show similar PM characteristics and to clarify each sport's distinct PM features.

Comparisons across laboratory/scenario-based, recorded-video-based, reconstructed, and live-competition-based approaches could clarify which PM characteristics generalize across contexts and which depend on sport-specific informational and action constraints. Such work would support the complementary use of these approaches without treating the evidence they provide as interchangeable.

## 3 Discussion: implications for construct validity and future research

To advance PM assessment research, it is necessary to understand the standpoint from which participants are asked to evaluate PM. Researchers and readers must also interpret findings while being aware of what can and cannot be inferred from data obtained through PM-related assessment approaches. Whether previous studies have sufficiently distinguished between these standpoints when interpreting their findings remains questionable. If this perspective is incorporated into future research, the positioning of PM-related assessment approaches may become clearer, enabling the development of more systematic research.

The preceding discussion is summarized in Table 1, whereas Figure 1 situates the human-based assessment approaches within a broader multilevel process of PM-related evidence. Figure 1 should be read as a conceptual map of the complementary levels of PM-related evidence and the possible linkages among objective event/performance dynamics, athlete appraisal, subjective PM experience, observer inference, and retrospective reconstruction, rather than as a taxonomy of research designs or a fixed causal sequence. Objective event/performance dynamics constitute the temporally unfolding event structure from which PM may emerge, be inferred, or be reconstructed. Table 1, in contrast, classifies human-based PM assessment approaches according to evaluator standpoint and ecological context. Objective event/performance dynamics are therefore not included as a row in Table 1 because they do not necessarily involve a human evaluator. Instead, they are represented in Figure 1 as a distinct level of PM-related evidence that can be compared with observer-inferred, vicarious or simulated, reconstructed, and actor-experienced PM.

Within the actor pathway shown in Figure 1, expectations function as central reference values in the athlete's embodied appraisal. Athletes do not evaluate a point, an error, or an unfolding performance sequence solely according to whether their team is currently leading, level, or behind. Rather, such events are appraised relative to goals and expectations established before competition and potentially updated as the competition unfolds. This interpretation is consistent with appraisal-based accounts of emotion in competitive sport, in which situational significance and goal-related expectancies are positioned as starting points for affective and physiological responses (38). These expectations may reflect the perceived relative strength of the two teams, recent performance, tactical plans, player availability, and available information about the current conditions of both the athlete's own team and the opponent. Consequently, the same scoreline or performance event may carry different implications for PM depending on whether it confirms, exceeds, or violates what the athlete expected to occur. For example, an even score may be experienced positively by a team that expected to be substantially disadvantaged, but negatively by a team that expected to dominate. Expectations should therefore be regarded as important reference values through which objective event and performance dynamics acquire psychological meaning.

Bodily states should likewise not be understood only as contextual inputs into this appraisal process. Existing bodily conditions, such as fatigue, tension, arousal, or perceived energy, may influence how athletes interpret the unfolding situation and perceive their available possibilities for action. At the same time, expectation-based appraisals of unfolding events may themselves generate affective and bodily responses, including changes in arousal, tension, fatigue perception, energization, and physiological activation. These responses may then become inputs into subsequent appraisals, perceived action possibilities, and PM experience. Thus, the relationship among objective events, expectation-based appraisal, bodily states, and subjective PM should be understood as recursive rather than as a fixed unidirectional sequence (38).

However, Figure 1 is intended as a conceptual map rather than a fully specified causal model. The proposed linkages among objective event dynamics, expectation-based appraisal, bodily states, and subjective PM should therefore be understood as theoretical propositions requiring further empirical examination.

Building on this framework, future research should not assume that objective event/performance dynamics and actor-experienced PM necessarily converge. Previous dynamic-systems research has already shown that PM-related psychological states may change nonlinearly and in a history-dependent manner in relation to score progression and unfolding performance patterns under relatively structured or experimentally constrained conditions (6, 10). However, the objective indicators relevant to PM are likely to differ according to the structure and tactical logic of each sport. In net/wall sports, where points and score differentials change frequently, scoring sequences may provide particularly salient information about unfolding performance dynamics. In invasion sports with relatively infrequent scoring, such as soccer and rugby, PM may instead be shaped by the quality, continuity, and tactical meaning of play. Even commonly used objective indicators such as ball possession do not carry a fixed performance or psychological meaning. For example, when a team deliberately adopts a low defensive block, reduced possession may reflect the intended tactical plan rather than deteriorating performance. If athletes perceive that the defensive structure is functioning effectively and that the match remains under control, a positive sense of PM may be maintained despite low possession. Conversely, the same level of possession may be experienced negatively if athletes feel increasingly pressured or unable to control the situation. Thus, the key research question is not whether subjective PM simply mirrors an objective performance indicator, but under what tactical, temporal, expectancy-related, and situational conditions objective dynamics and actor-experienced PM align, diverge, or exhibit temporal lags. Investigating both alignment and divergence may reveal how athletes attribute psychological meaning to objective events in relation to tactical intentions, goals, expectations, perceived controllability, and situational significance.

To summarize, this study clarifies how PM-related evidence differs according to evaluator standpoint and ecological context. Laboratory/scenario-based, recorded-video-based, reconstructed, and live-competition-based approaches provide complementary rather than interchangeable evidence. Future studies should therefore specify which level of PM-related evidence their methods access and delimit the conclusions that can be drawn from those data.

## Data Availability

The original contributions presented in the study are included in the article/Supplementary Material, further inquiries can be directed to the corresponding author.
